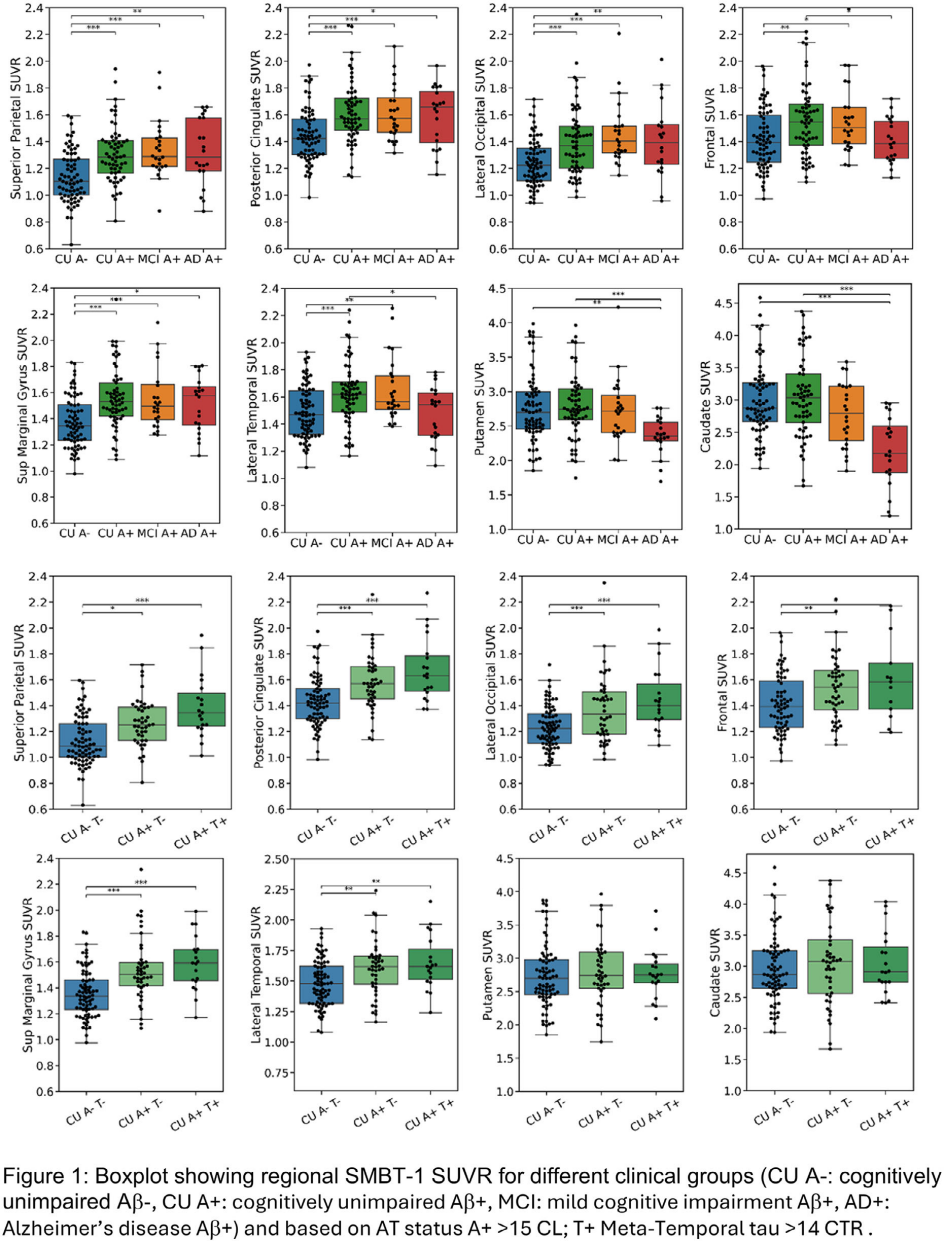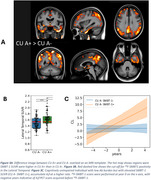# Reactive Astrogliosis predicts Amyloid Accumulation before the Preclinical Stage of Alzheimer's Disease

**DOI:** 10.1002/alz70856_105338

**Published:** 2026-01-07

**Authors:** Vincent Dore, Pierrick Bourgeat, Ryuichi Harada, Shozo Furumoto, Rachel S Mulligan, Ying Xia, Ishara Paranawithana, Simon M. Laws, Azadeh Feizpour, Svetlana Bozinovski, Kun Huang, Brian J Lopresti, Milos D. Ikonomovic, Jurgen Fripp, Nobuyuki Okamura, Christopher C. Rowe, Victor L. Villemagne

**Affiliations:** ^1^ Department of Molecular Imaging & Therapy, Austin Health, Melbourne, VIC, Australia; ^2^ CSIRO, Melbourne, VIC, Australia; ^3^ CSIRO, Brisbane, QLD, Australia; ^4^ Tohoku Medical and Pharmaceutical University, Sendai, Miyagi, Japan; ^5^ Research Center for Accelerator and Radioisotope Science, Tohoku University, Sendai, Miyagi, Japan; ^6^ Austin Health, Melbourne, VIC, Australia; ^7^ CSIRO Health and Biosecurity, Australian E‐Health Research Centre, Brisbane, QLD, Australia; ^8^ CSIRO Health and Biosecurity, Australian E‐Health Research Centre, Parkville, VIC, Australia; ^9^ Centre for Precision Health, Edith Cowan University, Joondalup, Western Australia, Australia; ^10^ The Florey Institute of Neuroscience and Mental Health, Parkville, VIC, Australia; ^11^ University of Pittsburgh, Pittsburgh, PA, USA; ^12^ University of Pittsburgh School of Medicine, Pittsburgh, PA, USA; ^13^ VA Pittsburgh Healthcare System, Pittsburgh, PA, USA; ^14^ Tohoku University Graduate School of Medicine, Sendai, Japan; ^15^ Institute of Development, Aging and Cancer, Tohoku University, Sendai, Japan; ^16^ Molecular Research and Therapy, Austin Health and University of Melbourne, Heidelberg, VIC, Australia; ^17^ Florey Department of Neuroscience and Mental Health, University of Melbourne, Parkville, VIC, Australia

## Abstract

**Background:**

Monoamine Oxidase‐B (MAO‐B) is overexpressed in reactive astrocytes, playing a crucial role in neurodegeneration. Recently, increased binding of the PET MAO‐B tracer ^18^F‐SMBT‐1 has been shown in preclinical Alzheimer's disease (AD) stages. However, the regional distribution and effect on Ab of abnormal ^18^F‐SMBT‐1 binding along the AD continuum remains unclear.

**Method:**

144 Cognitively Unimpaired (CU), 24 A+ Mild Cognitive Impairment (MCI), and 20 A+ AD subjects underwent PET imaging with ^18^F‐NAV4694, ^18^F‐MK6240, and ^18^F‐SMBT‐1.

Aβ and tau PET SUVR were transformed into Centiloid (CL) and CenTauR (CTR) using CapAIBL. A+ was defined as >15CL and T+ >14 CTR in the Meta‐Temporal. SMBT‐1 scans were spatially normalised using MR‐based CapAIBL, scaled to the cerebellar cortex and several cortical regions sampled. The relationship between SMBT‐1 binding and Aβ accumulation was assessed in a subset of 81 CU A‐.

**Result:**

Distinct regional ^18^F‐SMBT‐1 binding was observed across brain regions (Figure 1). ^18^F‐SMBT‐1 binding was higher in CU A+ compared to the CU A‐ in most regions, while binding in A+ MCI/AD either remained higher (in parietal/cingulate/occipital) or decreased (in frontal/caudate/putamen). Higher ^18^F‐SMBT‐1 binding was also significant in CU A+ T‐ (*n* = 43) (Figure 2). In CU A‐ participants with high ^18^F‐SMBT‐1 retention, significantly higher Ab accumulation rates were observed compared to low ^18^F‐SMBT1 (1.20CL/yr vs 0.01CL/yr, respectively, *p* = 0.001) (Figure 3). Furthermore, 92% of CU A‐ individuals with high ^18^F‐SMBT‐1 were classified as Ab accumulators (58% in CU A‐/low ^18^F‐SMBT‐1).

**Conclusion:**

Unlike fluid neuroinflammation markers, ^18^F‐SMBT‐1 facilitates quantitative assessment of regional differences in reactive astrogliosis across the AD continuum, as well as longitudinal change. Elevated ^18^F‐SMBT‐1 in CU A‐ predicted Ab accumulation, highlighting the potential of ^18^F‐SMBT‐1 as a prognostic marker in early‐stage AD while suggesting modulation of astrocytic function may be a target for AD prevention and treatment.